# Effects of mineral trioxide aggregate, Biodentine^TM^ and calcium hydroxide on viability, proliferation, migration and differentiation of stem cells from human exfoliated deciduous teeth

**DOI:** 10.1590/1678-7757-2016-0629

**Published:** 2018-01-16

**Authors:** Leandro Borges Araújo, Leopoldo Cosme-Silva, Ana Paula Fernandes, Thais Marchini de Oliveira, Bruno das Neves Cavalcanti, João Eduardo Gomes, Vivien Thiemy Sakai

**Affiliations:** 1 Universidade Federal de Alfenas, Faculdade de Odontologia, Departamento de Clínica e Cirurgia, Alfenas, Minas Gerais, Brasil; 2 Universidade de São Paulo, Faculdade de Odontologia de Bauru, Departamento de Odontopediatria, Ortodontia e Saúde Coletiva, Bauru, São Paulo, Brasil; 3 University of Iowa, College of Dentistry, Iowa City, Iowa, United States of America; 4 Univ. Estadual Paulista, Faculdade de Odontologia, Departamento de Odontologia Restauradora, Araçatuba, São Paulo, Brasil

**Keywords:** Biomaterials, Cell differentiations, Dental pulp capping, Stem cells, Vital pulp therapy

## Abstract

**Objective:**

The aim of the study was to evaluate the effects of the capping materials mineral trioxide aggregate (MTA), calcium hydroxide (CH) and Biodentine^TM^ (BD) on stem cells from human exfoliated deciduous teeth (SHED) *in vitro.*

**Material and Methods:**

SHED were cultured for 1 – 7 days in medium conditioned by incubation with MTA, BD or CH (1 mg/mL), and tested for viability (MTT assay) and proliferation (SRB assay). Also, the migration of serum-starved SHED towards conditioned media was assayed in companion plates, with 8 μm-pore-sized membranes, for 24 h. Gene expression of dentin matrix protein-1 (DMP-1) was evaluated by reverse-transcription polymerase chain reaction. Regular culture medium with 10% FBS (without conditioning) and culture medium supplemented with 20% FBS were used as controls.

**Results:**

MTA, CH and BD conditioned media maintained cell viability and allowed continuous SHED proliferation, with CH conditioned medium causing the highest positive effect on proliferation at the end of the treatment period (compared with BD and MTA) (p<0.05). In contrast, we observed increased SHED migration towards BD and MTA conditioned media (compared with CH) (p<0.05). A greater amount of DMP-1 gene was expressed in MTA group compared with the other groups from day 7 up to day 21.

**Conclusion:**

Our results show that the three capping materials are biocompatible, maintain viability and stimulate proliferation, migration and differentiation in a key dental stem cell population.

## Introduction

Vital pulp therapy based on the use of stem cell has promising research and therapeutic applications in dentistry. Stem cells in the dental pulp respond to tooth injuries by migrating, proliferating and differentiating into odontoblasts, which conduct the synthesis and secretion of tertiary dentin^6^ ^,^ ^9^ ^,^ ^17^ ^,^ ^32^. In particular, deciduous teeth contain a population of postnatal “stem cells from human exfoliated deciduous teeth” (SHED), which are capable of extensive proliferation and multipotential differentiation *in vitro* ^8^ ^,^ ^17^ ^,^ ^19^. In dental tissues, SHED constitute an autogenous source of cells because of their potential for pulp regeneration *in vivo,* which could be applied in vital pulp therapy^7^ ^,^ ^12^ ^,^ ^25^.

Following the diagnosis of deep carious/traumatic pulp injuries of primary and permanent teeth, vital pulp therapy may be clinically performed by applying a capping material directly onto the pulp tissue to allow pulp/dentine regeneration^2^ ^,^ ^12^. During conservative pulp treatments such as pulpotomy and direct pulp capping, interactions between pulp cells and the capping material affect stem cell proliferation^6^ ^,^ ^29^. While the exact nature of interactions between capping materials and the injured pulp tissue (during wound healing and regeneration) remains unclear, *in vitro* studies in two or three-dimensional culture systems as well as *in vivo* studies on capping materials have aided in material selection, which is the key to ensure a good treatment outcome^2^ ^,^ ^6^ ^,^ ^8^ ^,^ ^15^ ^,^ ^26^ ^,^ ^29^.

An ideal capping material should be highly biocompatible, prevent bacterial microleakage and promote the formation of mineralized tissue^16^ ^,^ ^18^ ^,^ ^24^. Different materials are used in the endodontic treatment of primary and permanent teeth, including calcium hydroxide (CH), mineral trioxide aggregate (MTA), and the recently launched Biodentine^TM^ (BD)^16^ ^,^ ^24^.

CH is composed of calcium ions, which react with the carbon dioxide present in tissues, producing calcite granules. This process leads to the accumulation of fibronectin, which allows cell adhesion and differentiation, thus resulting in the formation of mineralized tissue^4^. MTA and BD are hydraulic calcium silicate cements, which require moisture for hardening reaction^23^. They have the capacity to create a suitable bioactive surface with an appropriate architecture because of the nucleation of calcium phosphates and the formation of an apatite^23^. MTA has the capacity to maintain pulp vitality and promote healing when in contact with dental pulp or periradicular tissue^20^. The effect of MTA as a capping agent may also be seen in root canals, where an active mineralized tissue deposition and narrowing or obliteration of the canal occur^20^. BD stimulates cell differentiation and promotes mineralization in human dental pulp cells^14^ ^,^ ^21^. It has physico-mechanical properties superior to those of MTA and similar to those of dentin; it also has easier handling and shorter setting time than MTA^2^. Because of these features, MTA and BD have gained great attention for clinical applications, such as pulp capping, pulpotomy, apexogenesis, root-end apicoectomy and root perforations and resorptions. Considering that the nature of stem cell response elicited by the different capping materials has not been defined and, specially, the effect of the BD on SHED, a key stem cell population of deciduous teeth, has not been examined, the purpose of our study was to evaluate the effects of MTA, CH and BD on SHED proliferation, viability, migration and odontogenic-like phenotype differentiation *in vitro.*

## Material and methods

### Cell culture

SHED, isolated and characterized as previously described^19^, were maintained in alpha-MEM medium supplemented with 10% fetal bovine serum (FBS, Certified, Heat-inactivated) and 1% penicillin and streptomycin solution (culture medium components were from Gibco, Invitrogen, Grand Island, NY, USA). Cells were maintained at 37°C and 5% CO_2_ and split at a ratio of 1:3 when they reached 80% confluence. The medium was changed every two days. For all experiments, SHED at passages 4 to 8 were used.

### Conditioned media preparation

BD (Septodont, St-Maur-des-Fosses, Cedex, France), MTA (White MTA, Angelus, Londrina, PR, Brazil) and CH (Biodynamics, Ibiporã, PR, Brazil) were applied to cultures as “conditioned media”^27^ ^,^ ^31^, to avoid direct contact with cells.

BD and MTA were prepared according to the manufacturer's instructions, as follows. After sterilization of the glass slab and metal spatula, 1 spoon of MTA powder and 1 drop of distilled water were mixed for 30 seconds until the mixture was homogeneous and with a consistency similar to wet sand. For BD preparation, 5 drops of the liquid were poured into the capsule containing the powder. The capsule was closed, placed on a mixing device (Silamat S6, Ivoclar Vivadent, São Paulo, SP, Brazil) at a speed of 4,000 rotations/min., and mixed for 30 seconds. BD and MTA were mixed with the culture medium for a final concentration of 1 mg/mL, according to Slompo, et al.^27^ (2015) and Woo, et al.^31^ (2013). CH powder was diluted directly in culture medium, for a final concentration of 1 mg/mL.

Media were “conditioned” with capping materials for 1 h at 37°C, and then centrifuged at 45x g, for 5 minutes, at 4°C. Supernatants were filtered using 0.45 mm pore-size Millex^®^ syringe filter (EMD Millipore Corporation, Billerica, MA, USA), aliquoted and frozen at −20°C.

### Stimulation with capping material conditioned medium

For stimulation, SHED were seeded into 96-well plates (2.5×10^3^ and ~1×10^4^ cells/wells, for MTT and SRB assays, respectively; 200 μl growth medium/well), and allowed to attach overnight. The medium was completely replaced by MTA, CH or BD conditioned media at 37°C, for 1, 3, 5 and 7 days. Regular culture medium (without conditioning) and culture medium supplemented with 20% FBS were used as negative and positive controls, respectively.

### MTT assay

After stimulation with medium conditioned by the different capping agents, the medium was replaced by 200 μL/well of MTT (3-4,5-dimethylthiazol-2-yl-2,5-diphenyltetrazolium bromide; Sigma-Aldrich, St. Louis, MO, USA) solution (MTT concentration, 0.5 mg/mL). After a 4-hour incubation at 37°C, the supernatant was discarded, 200 μL dimethyl sulfoxide (DMSO, Fisher Scientific, Hampton, NH, USA) was added to each well, and the absorbance at 560 nm was read in an Anthos Zenyth 200 RT microplate reader (Biochrom, Holliston, MA, USA). All absorbance measurements were blank corrected. Data correspond to conditions performed in triplicates, in three independent experiments.

### SRB assay

After stimulation with medium conditioned by the different capping agents, cells were fixed by the addition of 10% trichloroacetic acid and incubated for 1 hour at 4°C. Plates were washed in tap water 5 times and allowed to dry. Cellular protein was stained by adding 4% SRB (sulforhodamine B) in 1% acetic acid and incubated at room temperature for 30 minutes. Excess SRB was removed by washing with 1% acetic acid and the remaining SRB was solubilized in 10 mM Tris-base (unbuffered). The absorbance at 565 nm was read in an Anthos Zenyth 200 RT microplate reader (Biochrom, Holliston, MA, USA). All absorbance measurements were blank corrected. Data correspond to conditions performed in triplicates, in three independent experiments.

### Cell migration assay

Each well of 24-well companion plates (Fisher Scientific, Pittsburgh, NA, USA) was loaded with 500 mL of medium conditioned with MTA, BD or CH, culture medium supplemented with 20% FBS (positive control) or regular culture medium (negative control, with 10% FBS). Twenty-four-hour serum-starved SHED (2×10^5^ cells/well) were seeded into 8 mm-pore-sized inserts (200 mL culture medium/well) and allowed to migrate toward the conditioned media overnight, at 37°C. Then, inserts were removed and migrated cells were trypsinized, spun in microcentrifuge tubes at 3,835x g at 4°C for 10 minutes, washed with PBS and spun again as previously. Cells in the final pellet were transferred to new 24-well plates containing 20 mM Cell Tracker^TM^ Green CMFDA (Invitrogen, Carlsbad, CA, USA) in PBS (500 mL/well). Plates were incubated in the dark (at room temperature) for 30 minutes, and fluorescence was read at 485 in an Anthos Zenyth 200 RT microplate reader (Biochrom, Holliston, MA, USA). All absorbance measurements were blank corrected. Data correspond to conditions performed in triplicates, in three independent experiments.

### Odontogenic-like phenotype differentiation by RT-PCR

SHED (2×10^3^) were cultured in 6-well plates with MTA, BD or CH conditioned medium for 1, 7, 14 and 21 days. Total RNA was extracted with Trizol reagent (Invitrogen, Carlsbad, CA, USA), according to the manufacturer's instruction, and RNA concentration was measured using Nanodrop 2000C spectrophotometer (Wilmington, DE 19810, USA).

RT-PCR was performed using 1 μg of RNA and SuperScript^®^ III One-Step RT-PCR System with Platinum^®^ Taq DNA Polymerase (Invitrogen, Carlsbad, CA, USA), according to the manufacturer's instruction. SHED differentiation was monitored by detection of dentin matrix protein-1 (DMP-1) gene expression (sense primer 5’-CAGGAGCACAGGAAAAGGAG-3’ and antisense primer 5’-CTGGTGGTATCTTGGGCACT-3’, expected product size: 213 bp). Glyceraldehyde-3-phosphate dehydrogenase (GAPDH) gene was used as a constitutive reference gene (sense primer 5’-GACCCCTTCATTGACCTCAACT-3’ and antisense primer 5’-CACCACCTTCTTGATGTCATC-3’, expected product size: 683 bp). The thermal cycling process consisted of reverse transcription for 30 minutes at 55°C and 2 minutes at 94°C, followed by 35 cycles of PCR amplification. Each cycle consisted of denaturation (94°C for 30 seconds), annealing (55°C for 30 seconds) and extension (72°C for 1 minute). Samples were incubated for an additional period of 10 minutes at 72°C (final extension) after the last cycle. An aliquot of 5 μl of each sample was analyzed by horizontal electrophoresis on a 2% agarose gel stained with ethidium bromide (0.64 mg/mL) at 60 V for 120 minutes. PCR products were compared with molecular weight marker with 100-bp intervals, visualized under ultraviolet light and photo-documented.

### Statistical analysis

Statistical analysis was performed using GraphPad Prism 7.01 software (GraphPad, UK). Viability, proliferation and migration data were analyzed by two- and one-way analysis of variance (ANOVA), followed by Tukey's test. Differences were considered significant at p<0.05.

## Results

**Figure 1:**
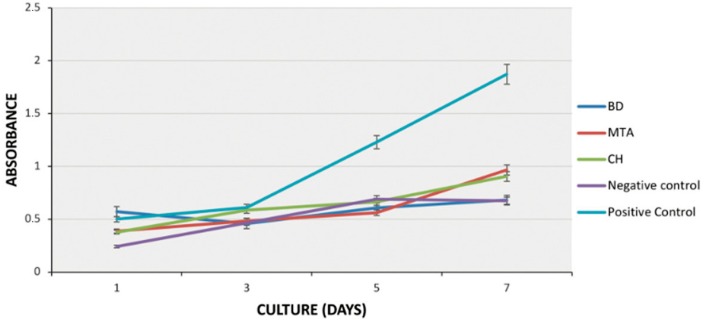
Effect of medium conditioned by calcium hydroxide (CH), mineral trioxide aggregate (MTA) or Biodentine^TM^ (BD) on the viability of stem cells from exfoliated deciduous teeth (SHED). Cells were incubated with conditioned media (1 mg/mL) for 7 days, at 37°C, and then subjected to the MTT assay. Regular growth medium (alpha-MEM with 10% FBS) or medium with 20% FBS were used as negative and positive control, respectively. Data represent mean ± SD (n=3 independent experiments, performed in triplicates)

To estimate the effect of different agents on cell viability, SHED were stimulated with CH (CH group), MTA (MTA group) or BD (BD group) conditioned medium for seven days, and subjected to the MTT assay. The viabilities of SHED treated with either of the three conditioned media were similar after 1, 3 and 5 days of treatment (p>0.05; Figure 1), and lower than that of the positive control after 3, 5 and 7 days (p<0.05). After 7 days of treatment, the viability of cells in the MTA group was similar to that in CH (p>0.05; Figure 1), but higher than that in the negative control and the BD group (p<0.05; Figure 1).

**Figure 2:**
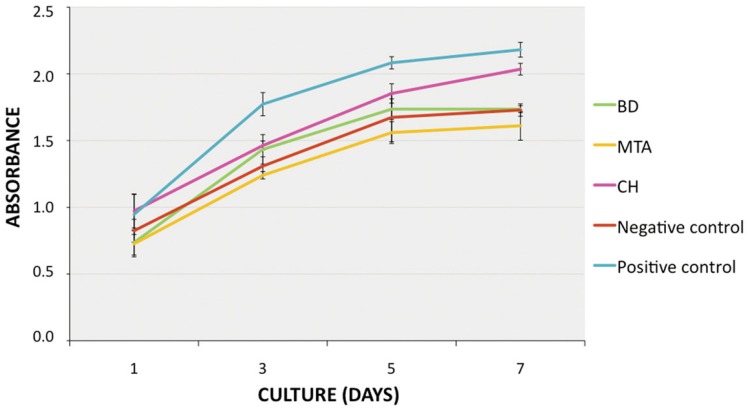
Effect of calcium hydroxide (CH), mineral trioxide aggregate (MTA) or Biodentine^TM^ (BD) conditioned medium on the proliferation of stem cells from exfoliated deciduous teeth (SHED). Cells were incubated with conditioned media (1 mg/mL) for 7 days, at 37°C, and then subjected to the SRB assay. Regular growth medium (alpha-MEM with 10% FBS) or medium with 20% FBS were used as negative and positive control, respectively. Data represent mean ± SD (n=3 independent experiments, performed in triplicates)

To examine the effect of conditioned media on SHED proliferation, SRB assay was used (Figure 2). A clear SHED proliferation in all treated groups was observed, although proliferation in the positive control was significantly higher than that in the negative control, CH, MTA and BD groups (p<0.05; Figure 2). After 24 hours of treatment, the proliferation of cells in the CH group was similar to that in positive control; in contrast, proliferation in the BD group was similar to that of the negative control. Cells stimulated with MTA had the lowest proliferation in the first day of evaluation (p<0.05). After 3 and 5 days of treatment, the CH and BD groups had similar proliferation (p>0.05), which was higher than that in the MTA group (p<0.05). After 7 days, cells in the CH group had significantly higher proliferation than those in the BD and MTA groups (p<0.05), reaching similar cell densities (p>0.05).

**Figure 3:**
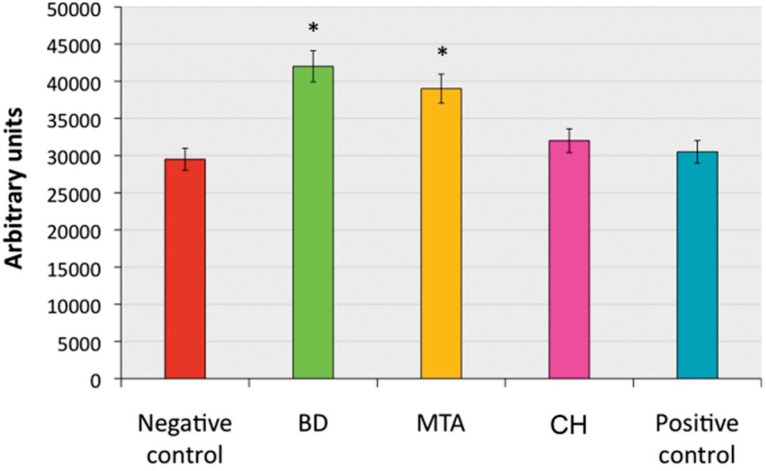
Migration of stem cells from human exfoliated deciduous teeth (SHED) toward medium conditioned by incubation with calcium hydroxide (CH), mineral trioxide aggregate (MTA) or Biodentine^TM^ (BD). Serum-starved SHED could migrate through 8-μm pore-size inserts (in companion plates) overnight, and the number of migrating cells was estimated by staining with Cell Tracker^TM^ Green CMFDA. Regular growth medium (alpha-MEM with 10% FBS) or medium with 20% FBS were used as negative and positive control, respectively. Data represent mean ± SD (n=3 independent experiments, performed in triplicates). *p<0.05 (one-way analysis of variance)

Similar levels of SHED migration towards CH conditioned media, and toward the positive and the negative controls were observed (p>0.05), while migration was significantly higher in the BD and MTA groups (p<0.05). No difference was observed between the migration towards BD and MTA conditioned media (p>0.05, Figure 3).

**Figure 4:**
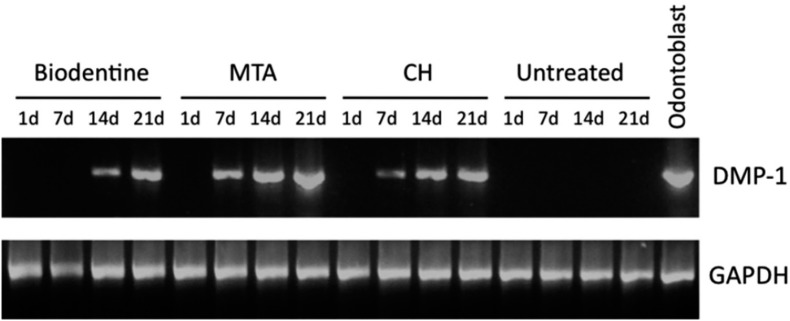
RT-PCR analysis of dentin matrix protein-1 (DMP-1, 213 bp) and glyceraldehyde-3-phosphate dehydrogenase (GAPDH, 683 bp) mRNA expression on stem cells from human exfoliated deciduous teeth (SHED) cultured with calcium hydroxide (CH), mineral trioxide aggregate (MTA) or Biodentine^TM^ (BD) conditioned medium or regular growth medium (alpha-MEM supplemented with 10% FBS, negative control) for 21 days. Positive controls were odontoblasts retrieved from freshly extracted human third molars

As represented in Figure 4, all the samples expressed the constitutive gene GAPDH. DMP-1 gene expression was observed when SHED were cultured with the three capping material conditioned media, but not in the negative control. Notably, earlier DMP-1 expression was detected when cells were treated with MTA and CH in comparison with those treated with BD. In addition, relatively up-regulated DMP-1 expression when SHED were cultured with MTA was seen on days 7, 14 and 21.

## Discussion

In this study, we examined the effect of the capping materials CH, MTA and BD on SHED, by incubating these cells with medium that had been exposed to each of these capping agents (“conditioned medium”). We preferred to use conditioned medium rather than direct incubation with capping agents to simulate a clinical situation in which the dental pulp stem cells residing near blood vessels and nerve fibers would receive the soluble factors released by the capping material^17^. As a positive control, cells were grown in medium with 20% FBS, while negative control cells were grown in regular growth medium, with 10% FBS.

Although dental pulp stem cells are expected to play a key role in pulp/dentin regeneration during pulp therapy, current knowledge on the nature of stem cell responses to capping materials is limited^1^ ^,^ ^5^ ^,^ ^8^. In this study, we showed that medium conditioned by incubation with 1 mg/mL of either of the three capping materials tested - CH, MTA or BD - maintained SHED viability and promoted the cell proliferation, migration and differentiation, compared with the negative control. However, the results varied according to the material tested.

BD and MTA are based on calcium silicates, but their chemical compositions have subtle differences that influence cellular responses. In addition, the nanoscale surface characterization is of great importance for better understanding their biocompatibility since the outermost chemical surface will be the layer in intimate contact with the tissues, controlling macromolecular and protein adsorption and desorption, further resulting in repair or necrosis^10^. Nevertheless, the interaction of cells with the active components of the material and the mechanisms responsible for cellular responses are not completely understood, thus requiring further studies to correlate the nanosurface composition with the biological response of the pulp cells.

The viabilities of SHED cultured in the MTA, CH or BD conditioned media were similar to or higher than those in the negative control at all time-points of treatment. In addition, SHED treated with either of the three conditioned media proliferated similarly to or more than those in the negative control, suggesting the lack of cytotoxicity of these materials at 1 mg/mL. In a histomorphological study using a rat pulp injury model, Tran, et al.^28^ (2012) reported similar rates of cell proliferation induced by MTA and BD, corroborating our results at day 7 of treatment. Luo, et al.^17^ (2014) observed that Biodentine^TM^ at 2 mg/mL and 0.2 mg/mL significantly increased the proliferation of human dental pulp stem cells, while treatment with Biodentine^TM^ at 20 mg/mL resulted in a marked decreased cell proliferation, suggesting that BD is cytotoxic at this concentration. Similarly, the marked decrease in cell viability after treatment of transfected human pulp cells and human tooth germ stem cells with CH reported in other studies may be attributed to the use of high CH concentrations^3^ ^,^ ^11^. Ji, et al.^14^ (2010) showed that the low-concentration CH has positive effects on the proliferation of postnatal dental stem cells, obtained from an immature dental tissue of beagle dogs, in agreement with our data. In another study, MTA stimulated higher cell proliferation than CH and the negative control^11^. These variable data between our results and those from other studies may be explained by experimental differences in conditioned media preparation and in the methodology of cell proliferation analysis.

Cell migration to areas with higher concentrations of chemoattractive agents is necessary for homeostatic tissue maintenance and the regeneration of injured tissues^17^ ^,^ ^30^. This study is the first to compare the effects of MTA, BD and CH as chemoattractants for SHED migration. Our migration data indicate that higher numbers of SHED migrate toward BD and MTA, compared to CH and positive control media. Curiously, the migration data are in contrast with those of cell proliferation, which was higher in the CH group. Zhu, et al.^31^ (2014) found that MTA induced significantly higher migration than the negative control in human bone marrow-derived mesenchymal stem cells and human dental pulp cells, respectively, which corroborates our results. Other authors suggested that BD can be used as a pulp-capping agent since this material exhibited similar biocompatibility, and triggered similar levels of inflammatory response and odontoblastic differentiation, compared with MTA, in human dental pulp cells^5^.

Odontoblast cells are essential for the formation of reparative dentin in an injured tooth. A non-cytotoxic material that stimulates the differentiation of stem cells into odontoblasts can be considered a good material for pulp capping. In this study, DMP-1 was selected as a differentiation marker for detecting odontogenic potential of SHED treated with different materials. DMP-1 is a gene expressed in early odontoblast differentiation and plays an important regulatory role in collagen matrix organization and dentin mineralization^21^. However, it is known that the evaluation of only one marker of differentiation is not sufficient to conclusively determine whether the resulting cells are true odontoblasts, which consisted a limitation of this study.

Cells treated with MTA, CH and BD showed a progressive increase in DMP-1 gene expression, reaching the highest intensity on day 21. Although the number of differentiated cells has not been quantified, the visual evaluation of band intensities suggested that MTA had the highest potential to induce DMP-1 gene expression followed by CH, both inducing DMP-1 expression on day 7. BD induced late DMP-1 expression since the bands were detected after 14 and 21 days of stimulation. In the work of Peng, et al.^22^ (2011), tricalcium silicate (main component of MTA and Biodentine^TM^) had greater odontogenic potential compared with CH observed through the expression pattern of odontogenic markers (DSPP, DMP-1, osteocalcin, alkaline phosphatase and collagen type I).

The bioactivity of proliferation, migration and differentiation of dental pulp stem cells is regulated by networks of signaling molecules, including growth factors and their, receptors and transcriptional control systems^13^. It is likely that differences in SHED responses to the various capping materials are due to differential modulation of growth factor release, signaling pathway activation and transcriptional regulation. While the evaluation of these factors is beyond the scope of this work, these are important aspects for future studies on the response of SHED to capping agents.

## Conclusions

The three capping materials - Biodentine^TM^, MTA and CH - maintained viability and stimulated proliferation, migration and odontogenic-like phenotype differentiation in SHED, which supports the notion that these materials are adequate for pulpotomy treatment of primary teeth, where SHED are autogenous sources for vital pulp therapy.
